# Freeze-Drying Technique for Microencapsulation of *Elsholtzia ciliata* Ethanolic Extract Using Different Coating Materials

**DOI:** 10.3390/molecules25092237

**Published:** 2020-05-09

**Authors:** Lauryna Pudziuvelyte, Mindaugas Marksa, Katarzyna Sosnowska, Katarzyna Winnicka, Ramune Morkuniene, Jurga Bernatoniene

**Affiliations:** 1 Institute of Pharmaceutical Technologies, Medical Academy, Lithuanian University of Health Sciences, Sukileliu pr. 13, LT-50161 Kaunas, Lithuania; lauryna.pudziuvelyte@lsmuni.lt; 2 Department of Drug Technology and Social Pharmacy, Medical Academy, Lithuanian University of Health Sciences, Sukileliu pr. 13, LT-50161 Kaunas, Lithuania; 3 Department of Analytical and Toxicological Chemistry, Medical Academy, Lithuanian University of Health Sciences, Sukileliu pr. 13, LT-50161 Kaunas, Lithuania; mindaugas.marksa@lsmuni.lt; 4 Department of Pharmaceutical Technology, Medical University of Białystok, Mickiewicza 2c, 15222 Białystok, Poland; katarzyna.sosnowska@umb.edu.pl (K.S.); kwin@umb.edu.pl (K.W.); 5 Department of Drug Chemistry, Medical Academy, Lithuanian University of Health Sciences, Sukileliu pr. 13, LT-50161 Kaunas, Lithuania; ramune.morkuniene@lsmuni.lt

**Keywords:** *Elsholtzia ciliata*, ethanolic extract, essential oil, freeze-drying, polyphenols, mucoadhesive properties

## Abstract

The present study reports on the encapsulation of *Elsholtzia ciliata* ethanolic extract by freeze-drying method using skim milk, sodium caseinate, gum Arabic, maltodextrin, beta-maltodextrin, and resistant-maltodextrin alone or in mixtures of two or four encapsulants. The encapsulation ability of the final mixtures was evaluated based on their microencapsulating efficiency (EE) of total phenolic compounds (TPC) and the physicochemical properties of freeze-dried powders. Results showed that the freeze-dried powders produced using two encapsulants have a lower moisture content, but higher solubility, Carr index, and Hausner ratio than freeze-dried powders produced using only one encapsulant in the formulation. The microencapsulating efficiency of TPC also varied depending on encapsulants used. The lowest EE% of TPC was determined with maltodextrin (21.17%), and the highest with sodium caseinate (83.02%). Scanning electron microscopy revealed that freeze-drying resulted in the formation of different size, irregular shape glassy particles. This study demonstrated good mucoadhesive properties of freeze-dried powders, which could be incorporated in buccal or oral delivery dosage forms. In conclusion, the microencapsulation of *E. ciliata* ethanolic extract by freeze-drying is an effective method to produce new value-added pharmaceutical or food formulations with polyphenols.

## 1. Introduction

Natural substances, polyphenols, have attracted attention of many investigators and from the wider society, due to their health benefits to humans, as they are known for their antioxidant [1], antibacterial [2], antiviral [3], anticancer [4], neuroprotective [5], and cardioprotective [6] activities. Based on these activities, polyphenols can be applied in food industry, pharmaceutical, and cosmetics areas. However, in some technological stages and during storage, polyphenols are affected by various factors, such as pH, high moisture content, high temperature, and the presence of light and oxygen, which could lead to the destruction of some primary compounds and an introduction of new compounds. These processes have an impact on polyphenols rich foods or organoleptic properties and biological activities of pharmaceutics [7,8]. Also, there are some other difficulties of using polyphenols in foods or pharmaceuticals. Some of them are insoluble in water and unstable when exposed to enzymes, which causes a loss of activity. High molecular weight polyphenols are difficult to absorb. Other polyphenols have a high rate of metabolism and are rapidly eliminated from the body [8]. To achieve the stabilization and protection of polyphenols, various encapsulation methods are used, and one of the most popular is freeze-drying. Encapsulation could maintain the pharmacological activities of polyphenols and prolong their shelf life. Additionally, microencapsulated phenols are easy to incorporate into food, pharmaceutical, and cosmetic products, because of their good physicochemical (flow ability, compression, mixing, density, and others) and organoleptic properties [8].

Freeze-drying is the most commonly used method of encapsulation [9] based on the dehydration by sublimation of a frozen sample [10]. Freeze-drying is a suitable microencapsulation technique for sensitive bioactive compounds [11], because substances are not exposed to high temperature as using spray-drying technique [12]. Freeze-dried products can be reconstituted quickly and easily, which is particularly valuable in case of emergency; for example, antibodies and vaccines, which need to be administered as quickly as possible. Also, freeze-drying technique is simpler than other microencapsulation techniques, because of the limited number of steps (comparing to, for example, coacervation, solvent extraction, supercritical fluid precipitation, and others).

The coating or carrier materials have an important role in the encapsulation process, since they may influence the efficiency of encapsulation and physicochemical properties, which impact the stability of freeze-dried powders [9,10]. Wall materials or encapsulants surround the active core materials during the microencapsulation process. Encapsulating agents may be selected from various types of natural or synthetic materials, like maltodextrins, modified starches, proteins, dextrins, and others [7,9,10]. These materials are suitable for microencapsulation, because of their ability to form films, viscosity and resistance to the gastrointestinal tract, solid content, biodegradability, safety, and low price [7]. The most common coating materials for encapsulation are maltodextrins [7,10]. High solubility, low viscosity, and optimal gel formation properties make maltodextrins suitable for microencapsulation of polyphenols using freeze-drying techniques [11,12,13]. Despite that, maltodextrins have high glycemic index, for this reason, microencapsulated products are not appropriated for people with diabetes or if they take low carbs diet. Beta-cyclodextrin is one of the most common materials used as encapsulating agent in freeze-drying [14]. Βeta-cyclodextrin has an ability to form inclusion complexes with other molecules by non-covalent bonding, and the complex stability increases with the electron-donor character of the substituents [14]. The inclusion complex could change physicochemical properties of molecules (solubility, taste and smell, volatility, release of bioactive compounds) [10]. However, beta-cyclodextrin has a poor solubility in cold water, and is also more expensive than maltodextrin. Proteins have potential coating effects, due to their film forming properties and suitable interactions with polyphenols [11]. Skim milk and sodium caseinate are utilized as coating agents in microencapsulation process by freeze-drying [15,16]. Gum Arabic is the most popular coating agent for polyphenols. It is easy to use gum Arabic alone or in mixtures with other encapsulants for its high solubility, surface-activity, low viscosity, good emulsifying ability, non-toxicity, and for being tasteless [9,17,18]. However, using gum Arabic as an encapsulant is expensive. Resistant-maltodextrin is a randomly linked alpha-glucoside oligosaccharide, which has a low glycemic index. Resistant-maltodextrin is a soluble dietary fiber, which has nutritional benefits. Researchers are focused on the favorable effects of resistant-maltodextrin on human health [19,20]. Resistant-maltodextrin is a suitable substance to be used as an encapsulating agent. There are few research works where resistant-maltodextrin was used as an encapsulant in spray-drying and freeze-drying techniques [21,22]. However, there are no publications testing resistant-maltodextrin as an encapsulation material for polyphenols obtained from the *Elsholtzia ciliata* herb by the freeze-drying method. *Elsholtzia ciliata* is an annual plant used as a spice and medicine in traditional China medicine. *E. ciliata* is native to Asia, but it grows in Europe, Africa, North America, South America, and India [23,24]. It is reported that about 33 species plants of the genus are distributed in China. *E. ciliata* also naturally grow in Lithuania. In Lithuania *E. ciliata* mostly used as a spice for cuisine or decoration, but it does not have a wide range of uses for health benefits. *E. ciliata* belongs to the *Lamiaceae* family, the most widely distributed family of plants. The enlarged *Lamiaceae* contains about 236 genera and 6900 to 7200 species [25]. The *Lamiaceae* family of plants are a rich source of biologically active compounds—their therapeutic effect is attributed to the presence of a wide range of secondary metabolites or phytochemicals, such as flavonoids, glycosides, alkaloids, saponins, terpenoids, and phenols, which have various pharmacological activities [26,27]. According to some publications, *E. ciliata* is a rich source of various biologically active compounds. The main compounds obtained in this plant are phenylpropanoids, terpenoids, phytosterols, polyphenols, ketones [23]. Wang, Gong, and Jiang [28] had identified the main volatile compounds of *E. ciliata* essential oil from different plant parts. Elsholtzia ketone, caryophyllene and 3-octanol were predominant compounds of essential oil produced from stem, leaf, and flower [28]. Pudziuvelyte et al. [24] obtained that dehydroelsholtzia ketone, elsholtzia ketone, sesquiterpenes caryophyllene, β-bourbonene, germacrene D, α-caryophyllene, and α-farnesene were the predominant compounds in the SPME composition of the frozen, fresh, and dried *E. ciliata* herbal samples. The main compounds of essential oil produced from dried herb were dehydroelsholtzia ketone (78.28%) and elsholtzia ketone (14.58%) [24].

Poplyphenols are other major group of active compounds determined in *E. ciliata* herb. Guo et al. [23] and Kim et al. [29] had determined caffeic acid, luteolin, apigenin, rosmarinic acid, and kumatakenin in *E. ciliata* extracts. Pudziuvelyte et al. [30] for the first time reported 13 new phenolic compounds obtained from *E. ciliata* ethanolic extracts, (neochlorogenic acid, quinic acid, chlorogenic acid, vitexin, *p*-coumaric acid, ferulic acid, luteolin-7-glucoside, luteolin-7-rutinoside, apigenin-7-glucoside, naringenin, procyanidin B, chrysin, and diosmetin).

According to a rich source of active compounds, *E. ciliata* herb could possess various beneficial effects for health. The scientific studies report that *E. ciliata* have antiviral, antibacterial [23], anti-inflammatory [23,31], antioxidant [23], anticancer [24], and vasorelaxing [30] activities. However, there is a lot of scientific interest in *E. ciliata*’s chemical composition and potential useful health effects. Also, it will be beneficial to extend the consumption of *E. ciliata* as a medicine in Lithuania and other countries.

Previously, *E. ciliata* ethanolic extract and essential oil were microencapsulated using a spray-drying technique [22]. In this study, the use of different coating materials achieved medium value physicochemical spray-dried properties, and the encapsulation efficiency of total phenolic compounds was not high [22]. The scientific interest to compare two encapsulation methods was needed. Additionally, there are no studies on the freeze-dried ethanolic *E. ciliata* extract or essential oil. Some advantages of microencapsulation using freeze-drying techniques are known and an expectation of this study is that the microencapsulation of *E. ciliata* biologically active compounds by freeze-drying techniques would make it possible to increase their bioavailability, producing new value-added pharmaceutical or food formulations with polyphenols, and protect them from environmental factors during storage.

The present study aimed for the selection of optimal encapsulants for freeze-dried powders to stabilize the concentration of polyphenols, and to reach suitable physicochemical parameters of powders. Sodium caseinate, skim milk, maltodextrin, beta-maltodextrin, resistant-maltodextrin, and gum Arabic were used as carriers for *E. ciliata* ethanolic extract and essential oil. Further, the moisture content, solubility, bulk and tapped volumes, morphology, and mucoadhesive properties were evaluated during experiments.

## 2. Results and Discussion

### 2.1. Influence of Wall Material Components on the Physicochemical Properties

Plants secondary metabolites polyphenols are useful biologically active substances for human health. Polyphenols possess various biological effects such as antioxidant, anti-inflammatory, antiviral, antibacterial, anticancer, and others. For this reason, herbs are used for prevention and treatment. However, herbal material and products produced from herbs are not always are effective enough because of low amounts and inactive compounds, which are very sensitive for environmental conditions, such as oxidation, pH, temperature, enzymes, and others. The negative effect of these conditions may increase degradation, reduce total amounts of active compounds in herbal preparations. To protect active compounds and to increase their potential positive effects for health, it is suitable to apply microencapsulation methods. A freeze-drying method for the microencapsulation of E. ciliata will be analyzed in this study.

**Figure 1:**
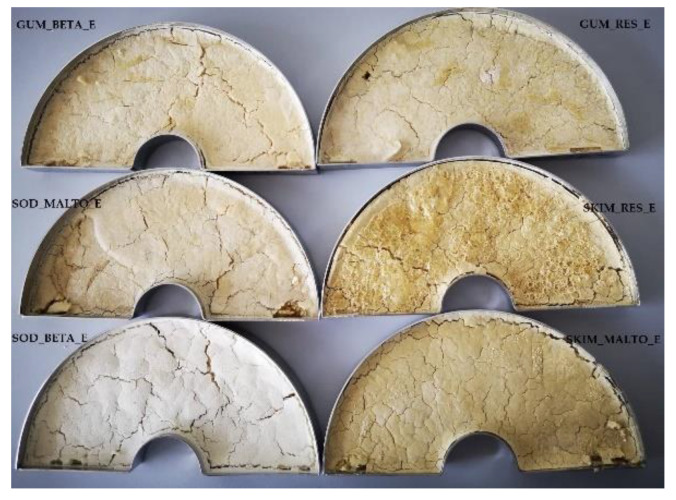
Samples of freeze-dried powders after freeze-drying.

At the early stage of the experiments, the most suitable encapsulant agent for core material microencapsulation using freeze-drying technique was selected. Six substances—skim milk, sodium caseinate, maltodextrin, resistant-maltodextrin, beta-cyclodextrin, and gum Arabic—were used as potential encapsulants. These coating materials were chosen according to their good properties, such as good solubility in water, low viscosity, ability to form films, resistance to gastrointestinal tract, solid content, biodegradability, safety, and low price. Also, maltodextrin and gum Arabic are the most commonly used encapsulants for microencapsulation. After freeze-drying, the physicochemical properties of freeze-dried powders (Figure 1) were analyzed.

**Figure 2:**
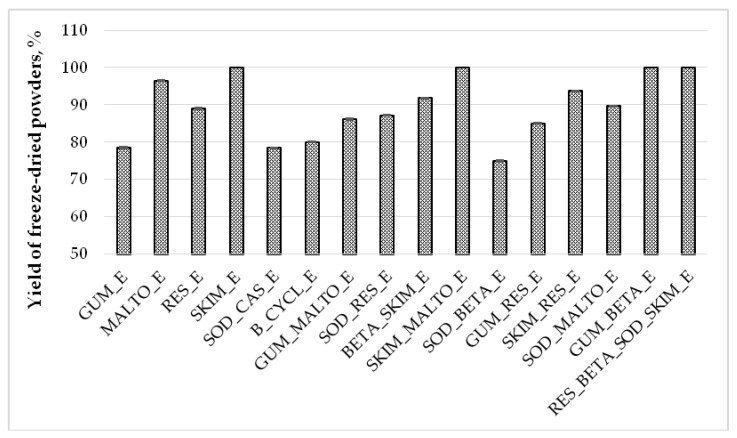
Yield of freeze-dried powders (%) obtained using different wall materials and mixtures. GUM_E—gum Arabic; MALTO_E—maltodextrin; RES_E—resistant-maltodextrin; SKIM_E—skim milk; SOD_CAS_E—sodium caseinate; B_CYCL_E—beta-cyclodextrin; GUM_MALTO_E—gum Arabic and maltodextrin; SOD_RES_E—sodium caseinate and resistant-maltodextrin; BETA_SKIM_E—beta-cyclodextrin and skim milk; SKIM_MALTO_E—skim milk and maltodextrin; SOD_BETA_E—sodium caseinate and beta-cyclodextrin; GUM_RES_E—gum Arabic and resistant-maltodextrin; SKIM_RES_E—skim milk and resistant-maltodextrin; SOD_MALTO_E—sodium caseinate and maltodextrin; GUM_BETA_E—gum Arabic and beta-cyclodextrin; RES_BETA_SOD_SKIM_E—resistant-maltodextrin, beta-cyclodextrin, sodium caseinate, and skim milk.

The yield of freeze-dried powders ranged from 75% to 100% (Figure 2). Statistically significant lowest yield of all freeze-dried powders was determined for SOD_BETA_E sample (*p* < 0.05) and the highest for these samples: SKIM_E, SKIM_MALTO_E, GUM_BETA_E, and RES_BETA_SOD_SKIM_E.

**Figure 3:**
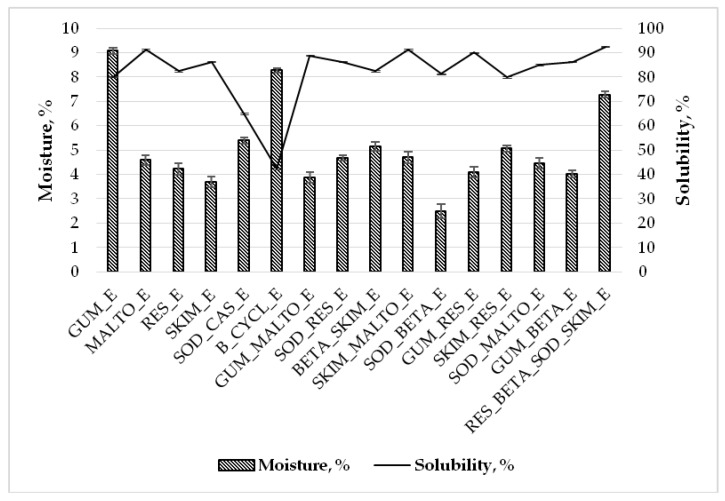
Moisture (%) and solubility (%) of freeze-dried powders obtained using different wall materials and mixtures. GUM_E—gum Arabic; MALTO_E—maltodextrin; RES_E—resistant-maltodextrin; SKIM_E—skim milk; SOD_CAS_E—sodium caseinate; B_CYCL_E—beta-cyclodextrin; GUM_MALTO_E—gum Arabic and maltodextrin; SOD_RES_E—sodium caseinate and resistant-maltodextrin; BETA_SKIM_E—beta-cyclodextrin and skim milk; SKIM_MALTO_E—skim milk and maltodextrin; SOD_BETA_E—sodium caseinate and beta-cyclodextrin; GUM_RES_E—gum Arabic and resistant-maltodextrin; SKIM_RES_E—skim milk and resistant-maltodextrin; SOD_MALTO_E—sodium caseinate and maltodextrin; GUM_BETA_E—gum Arabic and beta-cyclodextrin; RES_BETA_SOD_SKIM_E—resistant-maltodextrin, beta-cyclodextrin, sodium caseinate, and skim milk.

As Figure 3 shows, the moisture content of freeze-dried powders ranged from 2.49 ± 0.30% to 9.07 ± 0.12%. The lowest moisture content was found in the SOD_BETA_E sample (2.49 ± 0.30%), and the highest moisture content was in the GUM_E sample (9.07 ± 0.12%). As the results show (Figure 3), mixtures of two wall materials reduced freeze-dried powders moisture content compared to using only one substance as a wall material. The moisture content in the SOD_BETA_E sample was 2.49 ± 0.30%, SOD_CAS_E—5.39 ± 0.12%, and B_CYCL_E—8.27 ± 0.10%. GUM_E powders had the highest moisture content, but using gum Arabic in mixtures with maltodextrin, resistant-maltodextrin, and beta-cyclodextrin lowered the moisture content approximately two times (GUM_MALTO_E—3.88 ± 0.23%, GUM_RES_E—4.10 ± 0.21%, and GUM_BETA_E—4.01 ± 0.14%, respectively). Using freeze-drying as encapsulation method provides higher moisture content, as compared to spray-drying. The higher moisture for freeze-dried powders could be affected for the lower process temperature, compared to that which is using for spray-drying technique. Additionally, freeze-dried powders sometimes are more hygroscopic than spray-dried powders that will cause a higher moisture content. According to Kuck and Norena [7], spray-dried powders have three times lower moisture content than freeze-dried powders. The lower freezing temperature (−40 °C) results in a smaller pore size in the freeze-dried product due to higher cooling rate and increased nucleation [32]. Small pores resist mass transfer and act as a barrier against sublimation [32], retaining moisture in freeze-dried powder.

Using different wall materials impact the moisture content of freeze-dried powders. Data obtained by Ezhilarasi et al. [32] correspond to our study results, and show that using whey protein isolate and maltodextrin the moisture content is higher than using a mixture of these wall materials (15.65%, 12.56%, and 11.53%, respectively). Comparatively, whey protein isolate used in Ezhilarasi study and gum Arabic in our study increased moisture content, due to their potential to bind a great number of water molecules through hydrogen bonds. During the freezing process, the higher protein concentration in the solution may induce aggregation and make interstitial water less available for freezing [32].

An increased moisture content could negatively affect the freeze-dried powders during storage. Higher moisture content in the freeze-dried powders could reduce the quality of the powders, such as lower flowability, change the color, flavor, reduce amounts of predominant compounds, and their activity. Also, freeze-dried powders with high moisture content could be the perfect environment for microorganisms (bacterial contamination).

The solubility of the freeze-dried powders ranged from 42.50% to 92.50% (Figure 3). B_CYCL_E sample had the lowest solubility and the RES_BETA_SOD_SKIM_E freeze-dried powders sample had the highest. According to the data, mixtures of two wall materials increased solubility of freeze-dried powders. Using skim milk, sodium caseinate, and gum Arabic with beta-cyclodextrin in the mixtures, the solubility increased two times compared with the sample which contains only beta-cyclodextrin used as wall material (B_CYCL_E—42.50 ± 0.44%, BETA_SKIM_E—82.50 ± 0.32%, SOD_BETA_E—81.25 ± 0.34%, GUM_BETA_E—86.25 ± 0.24%). Using mixtures of sodium caseinate with resist-maltodextrin, beta-cyclodextrin, and maltodextrin, the solubility of freeze-dried powders increased (SOD_CAS_E—65.00 ± 0.36%, SOD_RES_E—86.25 ± 0.19%, SOD_BETA_E—81.25 ± 0.34%, and SOD_MALTO_E—85.00 ± 0.41%).

**Figure 4:**
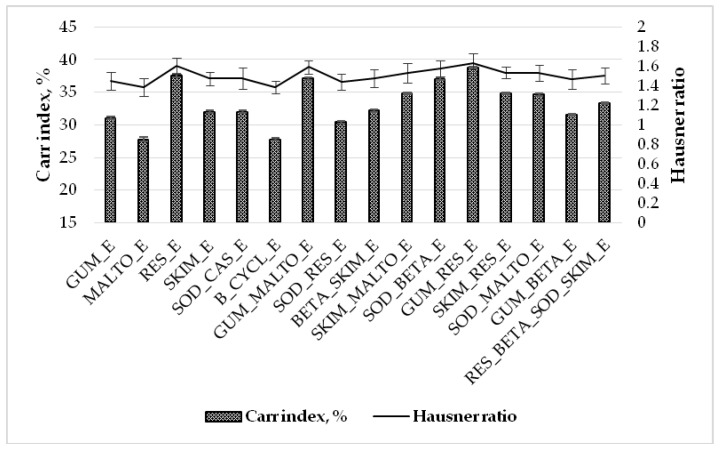
Carr index (%) and Hausner ratio of freeze-dried powders obtained using different wall materials and mixtures. GUM_E—gum Arabic; MALTO_E—maltodextrin; RES_E—resistant-maltodextrin; SKIM_E—skim milk; SOD_CAS_E—sodium caseinate; B_CYCL_E—beta-cyclodextrin; GUM_MALTO_E—gum Arabic and maltodextrin; SOD_RES_E—sodium caseinate and resistant-maltodextrin; BETA_SKIM_E—beta-cyclodextrin and skim milk; SKIM_MALTO_E—skim milk and maltodextrin; SOD_BETA_E—sodium caseinate and beta-cyclodextrin; GUM_RES_E—gum Arabic and resistant-maltodextrin; SKIM_RES_E—skim milk and resistant-maltodextrin; SOD_MALTO_E—sodium caseinate and maltodextrin; GUM_BETA_E—gum Arabic and beta-cyclodextrin; RES_BETA_SOD_SKIM_E—resistant-maltodextrin, beta-cyclodextrin, sodium caseinate, and skim milk.

The effects of different encapsulating agents on the Carr index and Hausner ratio of freeze-dried powders are shown in Figure 4. In the present study, the Carr index and Hausner ratio of freeze-dried powders ranged from 27.78% to 38.80% and 1.384 to 1.631, respectively, depending on the encapsulating agent (Figure 4). If the Carr index is less than 10%, this shows excellent flow. The low Carr index (11%–15%) indicates good flowability characteristics; while the relatively high Carr index (16–20%) and very high Carr index (>31%) indicate fair and very poor flowability characteristics [33].

The lowest Carr index and Hausner ratio were obtained for the MALTO_E and B_CYCL_E samples and the highest for the GUM_RES_E sample. The data shows that using mixtures of two or four wall materials increased Carr index and Hausner ratio values, which indicates that freeze-dried powders were characterized by poor flowability. As our study shows, using maltodextrin and beta-cyclodextrin alone is better for Carr index and Hausner ratio values than using maltodextrin and beta-cyclodextrin in mixtures with other substances (e.g., gum Arabic, skim milk, and sodium caseinate) (Figure 4). The flow rate of a material depends upon many factors related to the particle structure and processing conditions. The compressibility of a freeze-dried powder can affect its flow properties in the micro-scale through the adhesion forces between the particles. Coating materials have their individual properties of flowability. In the mixtures of two and more coating materials, the flowability could change because of interactions between encapsulants. Powders produced by a freeze-drying technique could possess poor flowability because of the high moisture content.

**Figure 5:**
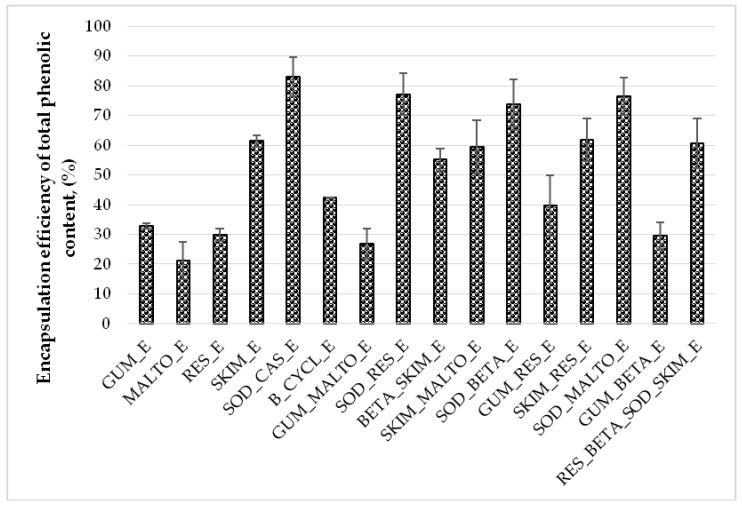
Encapsulation efficiency (EE, %) of total phenolic content (TPC, %) for freeze-dried powders obtained using different wall materials and mixtures. GUM_E—gum Arabic; MALTO_E—maltodextrin; RES_E—resistant-maltodextrin; SKIM_E—skim milk; SOD_CAS_E—sodium caseinate; B_CYCL_E—beta-cyclodextrin; GUM_MALTO_E—gum Arabic and maltodextrin; SOD_RES_E—sodium caseinate and resistant-maltodextrin; BETA_SKIM_E—beta-cyclodextrin and skim milk; SKIM_MALTO_E—skim milk and maltodextrin; SOD_BETA_E—sodium caseinate and beta-cyclodextrin; GUM_RES_E—gum Arabic and resistant-maltodextrin; SKIM_RES_E—skim milk and resistant-maltodextrin; SOD_MALTO_E—sodium caseinate and maltodextrin; GUM_BETA_E—gum Arabic and beta-cyclodextrin; RES_BETA_SOD_SKIM_E—resistant-maltodextrin, beta-cyclodextrin, sodium caseinate, and skim milk.

The EE% TPC of freeze-dried powders are shown in Figure 5. The TPC EE% of freeze-dried powders varied from 21.17% to 83.02%. The lowest TPC EE% was determined in the MALTO_E sample and the highest in the SOD_CAS_E. Data shows that mixtures of two encapsulants affecting the EE% of TPC for freeze-dried powders. Statistically significant higher EE% of TPC were obtained in freeze-dried powders, which had proteins, such as skim milk and sodium caseinate in their composition. Maltodextrin alone in a MALTO_E sample showed 21.17% EE of TPC, when in samples SKIM_MALTO_E and SOD_MALTO_E the EE% of TPC increases 2.8 and 3.6 times (59.39% and 76.46%), respectively (*p* < 0.05). The same effect was obtained using resistant-maltodextrin in composition with sodium caseinate and skim milk. RES_E sample which contains only resistant-maltodextrin determined 29.85% EE% of TPC, when samples SKIM_RES_E and SOD_RES_E possessed 2 and 2.5 times higher values EE% of TPC (61.79% and 77.13%), respectively (*p* < 0.05). According to the data, there was no statistically significant differences using dextrins and gum Arabic in the same compositions (*p* > 0.05). For example, using gum Arabic alone on the GUM_E sample the EE% of TPC was 32.73%, when using gum Arabic in composition with maltodextrin (GUM_MALTO_E), resistant-maltodextrin (GUM_RES_E), and beta-cyclodextrin (GUM_BETA_E) the values of EE% of TPC were 26.83%, 39.79%, and 29.62%, respectively.

The freeze-dried product microencapsulated with sodium caseinate (SOD_CAS_E) demonstrated an exceptional conservation of phenols and had the highest EE% of all the freeze-dried samples. Good microencapsulation of polyphenols using sodium caseinate could be because of perfect emulsifier, gelation properties. Caseins according to various studies have been shown to protect their contents against cold (storage and freeze-drying), oxidation, heat, UV radiation [11,15,16]. Sodium caseinate is a suitable substance for the microencapsulation of phenols. It increased the ability of maltodextrin and resistant-maltodextrin to encapsulate biologically active substances, and the results were higher than using only maltodextrin and resistant-maltodextrin. However, there were no significant differences using sodium caseinate in the mixture with other wall materials (beta-cyclodextrin) for EE of TPC. Similar results were achieved by Šaponjac et al. [8]. When soy proteins and whey for sour cherry pomace encapsulation by freeze-drying was utilized, EE was 94.90% and 90.10%, respectively).

Using different wall materials or mixtures impacts EE% of TPC. According to Papoutsis et al. [11], encapsulation productivity was higher when using maltodextrin with soybean protein (74.84 ± 1.05%) than maltodextrin with ι-carrageenan (58.46 ± 3.02%). The study by Hussain et al. [34] has shown that by using different wall materials for freeze-drying process, the TPC ranged from 94.28% to 68.22%. The highest amounts of TPC were obtained in samples containing 5% gum Arabic with 5% maltodextrin, and 10% gum Arabic [34]. The study by Šturm et al. [9] has shown that using different ratio of core material and encapsulants may impact EE% of TPC. The highest EE% of TPC was obtained using 1:3, 1:4, and 1:7 ratios of propolis and gum Arabic (56.80 ± 0.80%, 64.70 ± 1.90%, 45.3 ± 1.10%), respectively. Inulin, maltodextrin, and gum Arabic were used alone as encapsulants for propolis microencapsulation (1:10) by freeze-drying [9]. Statistically significant differences were obtained between inulin and gum Arabic (13.1 ± 1.3% and 31.3 ± 4.1%, respectively) and maltodextrin and gum Arabic (14.9 ± 0.4% and 31.3 ± 4.1%, respectively) used as coating materials [9]. These data show that different coating materials affect the EE% of TPC in freeze-dried powders. One of the main factors that could impact the amount of total phenols in freeze-dried powders is the formation of microparticles during freeze-drying, due to scattering of the substances inside the configuration of encapsulants [34].

### 2.2. Morphology

**Figure 6:**
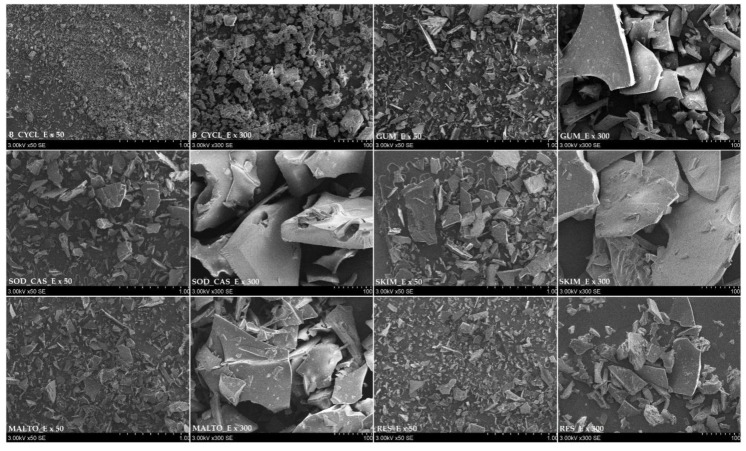
Images of freeze-dried powders obtained using different wall materials and mixtures. GUM_E—gum Arabic; MALTO_E—maltodextrin; RES_E—resistant-maltodextrin; SKIM_E—skim milk; SOD_CAS_E—sodium caseinate; B_CYCL_E—beta-cyclodextrin.

Structural analysis of the freeze-dried powders was conducted by scanning electron microscope (SEM). A comparison of the images showed a notable variation in terms of particle structure and size allotment amongst different microencapsulated products. All images of freeze-dried powders presented an irregular shape like broken glass, with some pores on surface (Figure 6). The structure of all the samples presented as uneven and brittle. Particles of the B_CYCL_E sample look smaller than in other samples of freeze-dried powders. Differences in surface area are also seen in SEM images. Images of GUM_E, MALTO_E, RES_E, SKIM_E, and SOD_CAS_E samples are quite similar—all particles have smooth surface area. However, images of the B_CYCL_E sample have a different look to the microparticles—a rough surface area. The difference in particle size may be associated with the type of wall material and the crushing of freeze-dried powders after the freeze-drying process.

### 2.3. Mucoadhesive Analysis

Mucoadhesive analysis of freeze-dried powders was performed to evaluate the potential use of the powders in buccal or oral delivery dosage forms. Two samples with the highest EE of TPC and sample, which contains four encapsulants, were chosen for the mucoadhesive test. Mucoadhesive properties of freeze-dried powders are presented in Table 1. All samples adhered to tested materials, and mucoadhesive properties were considerably (*p* < 0.05) influenced by the type of adhesive layer and composition of freeze-dried powders. Using a gelatin disc and porcine buccal mucosa, values of detachment force (F_max_) varied from 0.147 N to 0.390 N and from 0.085 N to 0.444 N. In the case of porcine buccal mucosa, the highest work of mucoadhesion (W_ad_) value 0.086 was observed for the RES_BETA_SOD_SKIM_E sample of freeze-dried powders. The lowest W_ad_ value using porcine buccal mucosa was determined for the SOD_BETA_E sample. A type of material used for the preparation of freeze-dried powders affected the adhesion, which was the highest for the RES_BETA_SOD_SKIM_E sample. SOD_CAS_E used alone in the composition of freeze-dried powders presented higher adhesion than used in a mixture with beta-cyclodextrin. Porcine stomach and buccal mucosa are valuable models of the adhesive membrane, due to their similarity to human mucosa in terms of histology, ultrastructure, and composition; they can be used to mimic the behavior of dosage forms in vivo [35]. Statistically significant differences were obtained for value of W_ad_ for all three samples using porcine buccal mucosa model (*p* < 0.05). The highest value of W_ad_ was determined for RES_BETA_SOD_SKIM_E sample (0.086 ± 0.003 µJ). When porcine stomach mucosa was used, F_max_ values ranged from 0.173 N to 0.444 N. Statistically significant differences for the values of W_ad_ were obtained between all three samples (*p* < 0.05). According to the results, the RES_BETA_SOD_SKIM_E sample obtained the highest value of W_ad_ (0.075 ± 0.007 µJ) compared to the SOD_CAS_E and SOD_BETA_E samples. In an acidic environment, freeze-dried powders with SOD_CAS_E presented lower adhesion to the mucous membrane than freeze-dried powders with SOD_BETA_E (0.047 ± 0.007 and 0.058 ± 0.004 µJ, respectively). This might be caused by the poor swelling of powders in acidic or/and neutral environment. The best mucoadhesive properties were noted when a mixture of four encapsulants (RES_BETA_SOD_SKIM_E) was utilized in the freeze-drying process.

## 3. Materials and Methods

### 3.1. Materials

Dried *E. ciliata* (Thunb.) Hyl were obtained from “Zolynu namai”, Vilnius, Lithuania. Dried herb was ground using Ultra Centrifugal Mill ZM 200 (Retsch, Haan, Germany). Grinding was performed at 6000 rpm using 0.25 mm trapezoid holes sieve.

Resistant-maltodextrin (Promitor 85™) was purchased from Bang & Bonsomer, (Vilnius, Lithuania), gum Arabic, skim milk, maltodextrin, sodium caseinate, beta-cyclodextrin were purchased from Sigma-Aldrich, (Steinheim, Germany). Ethanol (96%) used for extraction was purchased from Vilniaus degtine (Vilnius, Lithuania). All the chemicals used were of analytical grade.

### 3.2. Microencapsulation of E. ciliata Ethanolic Extract

Ethanolic *E. ciliata* extract was prepared by ultrassound-assisted extraction method, and essential oil by hydrodistillation, as described in a previous study [22]. Dried powdered *E. ciliata* herb was extracted 1:20 with 70% (*w*/*v*) ethanol in a conical flask using an ultrasound bath (Bandelin electronic GmbH & Co.KG, Berlin, Germany) at 25 °C for 30 min. Essential oil was obtained using Clevenger distillation apparatus. A dried grounded herb (30 g) was mixed with 500 mL purified water and submitted to extraction for 4 h at 120 °C.

Further, ethanolic extract and essential oil of *E. ciliata* were encapsulated using six different wall materials and their combinations: gum Arabic (GUM_E), maltodextrin (MALTO_E), resistant-maltodextrin (RES_E), skim milk (SKIM_E), sodium caseinate (SOD_CAS_E), beta-cyclodextrin (B_CYCL_E), gum Arabic and maltodextrin (GUM_MALTO_E); sodium caseinate with resistant-maltodextrin (SOD_RES_E), beta-cyclodextrin with skim milk (BETA_SKIM_E), skim milk with maltodextrin (SKIM_MALTO_E), sodium caseinate with beta-cyclodextrin (SOD_BETA_E), gum Arabic with resistant-maltodextrin (GUM_RES_E), skim milk with resistant-maltodextrin (SKIM_RES_E), sodium caseinate with maltodextrin (SOD_MALTO_E), gum Arabic with beta-cyclodextrin (GUM_BETA_E); and resistant-maltodextrin with beta-cyclodextrin, sodium caseinate, and skim milk (RES_BETA_SOD_SKIM_E). A sum of 20% (*w*/*v*) of each single encapsulant was mixed with purified water (in combinations of two encapsulants, 10% of each was added except RES_BETA_SOD_SKIM_E sample (0.54 g of sodium caseinate, 10 g of skim milk, 8.96 g of resistant-maltodextrin, and 0.5 g beta-cyclodextrin) at 22–25 °C and left for 12 h. After that, all the mixtures were stirred using magnetic stirrer (MSH-20A, Witeg, Wertheim, Germany) for 30 min at 25 °C. The solutions with dissolved encapsulants were mixed with *E. ciliata* ethanolic extract (50 mL) and essential oil (10 µL) mixture. All the prepared mixtures were homogenized for 5 min at 4000 rpm using IKA T18 digital Ultra-Turrax homogenizer (Staufen, Germany). The mixtures were frozen in the laboratory freezer FORMA™ 88,000 Series (Thermo Scientific, Waltham, MA, USA) at −80 °C for 24 h before the freeze-drying process. Finally, frozen samples were freeze-dried using laboratory freeze-dryer (LyoQuest Telstar, Wertheim, Germany) at −50 °C 0.05 mbar for 24 h. The freeze-dried powders were collected, packed in foil bags and stored in a dessicator prior to other analysis.

### 3.3. Moisture Content

The moisture content of the freeze-dried powders was measured by estimating the powder’s weight loss after oven drying at 105 °C, until a constant weight was obtained [36].

### 3.4. Solubility

According to Antonio et al. [37] method with some modifications the solubility of freeze-dried powder was determined. One gram of the sample was mixed with 25 mL of purified water for 5 min, using a magnetic stirrer MSH-20A (Witeg, Germany) at 300 rpm (25 °C). The mixture was transferred to a tube and centrifuged at 3000× *g* for 10 min at 25 °C, using centrifuge SIGMA3-18KS (Steinheim, Germany). A total of 20 mL of supernatant was transferred to a pre-weighed Petri dish and dried overnight in an oven at 105 °C. The solubility (%) of freeze-dried powder was calculated as the percentage of dried supernatant in relation to the amount of microcapsules by the equations:

(1) $$\mathrm{Solubility}\;=\frac{\mathrm{Residue}\;\mathrm{after}\;\mathrm{drying}}{\mathrm{Theoretical}\;\mathrm{residue}\;\mathrm{after}\;\mathrm{drying}}\times \;100\%$$

(2) $$\mathrm{Theoretical}\;\mathrm{residue}=\frac{W_{\mathrm{supernatant}\;\mathrm{to}\;\mathrm{be}\;\mathrm{dried}}-W_{\mathrm{microcapsules}}}{W_{\mathrm{microcapsules}}-W_{\mathrm{purified}\;\mathrm{water}}}$$

where W—weight.

### 3.5. Bulk and Tapped Volumes

The bulk and tapped density (V_0_ and V_tapped_) of freeze-dried powders were investigated using the density tester (SVM 102 Erweka, Langen, Germany), according to the Caliskan and Dirim [36] method, with some modifications. Ten grams of freeze-dried powders was weighed into a 100 mL measuring cylinder and tapped 500 times. Obtained values were then used to calculate Carr index and Hausner ratio:

(3) $$\mathrm{Carr}\;\mathrm{index}\;(\%)=\frac{100\;\times {\;(V}_{0}-{\;V}_{\mathrm{tapped}})}{V_{0}}$$

(4) $$\mathrm{Hausner}\;\mathrm{ratio}=\frac{V_{0}}{V_{\mathrm{tapped}}}$$

### 3.6. Total Phenolic Content (TPC) and Surface Phenolic Content (SPC) Determination

The total phenolic and surface phenolic contents were determined according to the methods of Tolun, Altintas, and Artik [33] with some modifications. A sum of 100 mg of freeze-dried powders were weighed and dissolved in 1 mL ethanol:acetic acid:water solution (20:8:42, *v*/*v*). The mixture was stirred using a magnetic stirrer for 1 min and ultrasonic bath for 20 min at 25 °C. After that, the mixture was filtered through a micro filter (0.45 µm). A sum of 100 µL of the sample and 2.5 mL of Folin-Ciocalteau reagent were mixed in a tube and left in the dark place for 5 min. Then, 2 mL of 7.5% sodium carbonate solution was added into the tube, mixed and left in the dark place for 1 h at 25 °C. TPC was expressed as mg equivalent of gallic acid per gram of freeze-dried powders. The absorbance was measured at 760 nm using a UV/VIS 1800 Shimadzu spectrophotometer (Shimadzu, Japan). For the determination of SPC of the freeze-dried powders, a 100 mg of sample was mixed with 10 mL of ethanol:methanol solution (1:1, *v*/*v*), and then filtered through a micro filter (0.45 µm). The SPC was obtained using the same method described for TPC determination. The SPC and TPC encapsulation efficiency (EE) were calculated according to Equations (5) and (6), respectively.

(5) $$\mathrm{SPC}\;\left(\%\right)=\frac{\mathrm{surface}\;\mathrm{phenolic}\;\mathrm{compounds}}{\mathrm{total}\;\mathrm{phenolic}\;\mathrm{compounds}}\times 100$$

(6) TPC EE (%) = 100 − SPC (%)

### 3.7. Morphology Analysis

The morphological characteristics of the freeze-dried powders were examined using scanning electron microscopy (Hitachi TM 3000, Tokyo, Japan). A small amount of freeze-dried powder sample was placed on the specimen holder. Images with magnifications of 50× and 300× were recorded at 3 kV.

### 3.8. Mucoadhesive Properties

Evaluation of the mucoadhesive properties was performed using TA.XT.Plus Texture Analyser (Stable Micro Systems, Godalming, United Kingdom), according to the Szekalska et al. method [35]. Porcine buccal and stomach mucosas, and gelatin discs were used as different models of adhesive layers. Experimental parameters of the mucoadhesive process were chosen during pilot tests and set as follows: applied force 1 N, pretest speed 0.5 mm/s, test speed 0.1 m/s, contact time 90 s, and posttest 0.1 mm/s. Discs of gelatin were prepared using a 30% (*w*/*w*) aqueous solution. Adhesive layers were adhered to an upper probe and moisturized with 0.1 M HCl (pH = 1.2) (stomach mucosa) and salive (pH = 6.8) (buccal mucosa). The tests were performed at 37 ± 1 °C. The mucoadhesive characteristics were obtained as the maximum detachment force (F_max_) and the work of mucoadhesion (W_ad_), calculated from the area under the force versus distance curve, expressed in µJ.

### 3.9. Statistical Analysis

One-way analysis of variance (ANOVA) followed by Tukey’s multiple comparison tests were performed using the software SPSS Statistics 20.0 (IBM Corporation, Armonk, NY, USA) to determine the significant (*p* < 0.05) differences between samples.

## 4. Conclusions

In this study, an ethanolic *E. ciliata* herb extract was successfully encapsulated by freeze-drying technique using skim milk, maltodextrin, sodium caseinate, gum Arabic, resistant-maltodextrin, and beta-cyclodextrin as encapsulants. The physicochemical properties and encapsulation efficiency of polyphenols were quite dependent on the wall material used. Different encapsulants impacted various properties of freeze-dried powders: moisture content, solubility, the Carr index, the Hausner ratio, morphology of particles, and encapsulation efficiency. Results showed that mixtures of two wall materials reduce moisture content, increase solubility, Carr index, and the Hausner ratio of freeze-dried powders, as compared to individual wall materials. The morphology of particles was quite similar in all samples, but there were slight differences in their form and size. The highest value of encapsulation efficiency of TPC was obtained for samples prepared using sodium caseinate alone or in mixture with resistant-maltodextrin and maltodextrin. According to mucoadhesive analysis results, the mixture of four encapsulants (RES_BETA_SOD_SKIM_E) presented stronger adhesion to buccal and stomach mucosa, as compared to the SOD_CAZ_E and SOD_BETA_E samples.

This data showed that freeze-drying is a suitable method for encapsulation of *E. ciliata* ethanolic extract and that the obtained freeze-dried powders contain high levels of polyphenols. The method and formulations of freeze-dried powders are appropriate for use in the pharmaceutical, cosmetics, or food industries. Freeze-dried powders could be incorporated in solid pharmaceutical form like hard capsules or tablets.

## Figures and Tables

**Table 1:** Mucoadhesive properties of freeze-dried powders.

Formulation	Kind of Adhesive Material					
	Gelatin Disc		Porcine Buccal Mucosa		Porcine Stomach Mucosa	
	F_max_ (N) ^1^	W_ad_ (µJ) ^2^	F_max_ (N) ^1^	W_ad_ (µJ) ^2^	F_max_ (N) ^1^	W_ad_ (µJ) ^2^
Control ^3^	0.038 ± 0.003	0.007 ± 0.001	0.057 ± 0.006	0.016 ± 0.002	0.031 ± 0.003	0.005 ± 0.001
SOD_BETA_E	0.390 ± 0.01	0.049 ± 0.006 ^a^	0.444 ± 0.015	0.058 ± 0.004 ^a^	0.444 ± 0.01	0.058 ± 0.004 ^a^
SOD_CAS_E	0.251 ± 0.045	0.046 ± 0.004 ^a^	0.2687 ± 0.01	0.074 ± 0.003 ^b^	0.173 ± 0.02	0.047 ± 0.007 ^b^
RES_BETA_SOD_SKIM_E	0.147 ± 0.01	0.047 ± 0.008 ^a^	0.085 ± 0.007	0.086 ± 0.003 ^c^	0.273 ± 0.02	0.075 ± 0.007 ^c^

^1^ Maximum detachment force, ^2^ work of adhesion, ^3^ cellulose paper used as a control; ^a, b, c^ in the columns of W_ad_ show statistically significant differences between samples of freeze-dried powders.
